# Associations of Sleep Duration and Physical Activity with Academic and Physical Education Performance Among Moroccan Adolescents

**DOI:** 10.3390/clockssleep8030055

**Published:** 2026-09-05

**Authors:** Anass Akhittouch, Hamid El Oirdi, Aziz Chokri, Mohamed Barkaoui, Said Ihbour, Aziz Eloirdi

**Affiliations:** 1 Sports Science Research Team, Institute of Sports Science, Hassan 1st University, Settat 26000, Morocco; hamid.eloirdi@usmba.ac.ma (H.E.O.); aziz.chokri@uhp.ac.ma (A.C.); mohamed.barkaoui@uhp.ac.ma (M.B.); said.ihbour@uhp.ac.ma (S.I.); 2 Laboratory of Biotechnology, Environment, Agri-Food and Health (BEAS), Faculty of Sciences Dhar El Mahraz, Sidi Mohamed Ben Abdellah University, Fez 30000, Morocco; 3 Institute of Sport Sciences, Sidi Mohamed Ben Abdellah University, Fez 30000, Morocco

**Keywords:** sleep duration, physical activity, academic performance, physical education, adolescents, Morocco, school performance

## Abstract

Background/Objectives: Sleep duration and physical activity are important lifestyle behaviors that may influence adolescents’ academic and Physical Education (PE) performance. However, evidence regarding their associations with Physical Education (PE) performance remains limited, particularly in North African populations. This study aimed to examine the relationships between sleep duration, physical activity, and academic and PE performance among Moroccan adolescents. Methods: A cross-sectional study was conducted among 439 Moroccan adolescents aged 14–20 years. Data were collected using the Arab Teens Lifestyle Study (ATLS) questionnaire. Sleep duration, physical-activity level (metabolic equivalent minutes per week [MET-min/week]), overall academic average, average grade in science subjects, and final PE grade were assessed. Pearson correlation analyses and multivariate analysis of variance (MANOVA) with follow-up univariate analyses were performed, with Games–Howell post hoc comparisons used for PE performance. Results: Sleep duration was positively associated with overall academic average (r = 0.147, *p* = 0.002) and final PE grade (r = 0.167, *p* = 0.001), but not with average grade in science subjects (r = 0.080, *p* = 0.099). Physical activity was positively associated with overall academic average (r = 0.169, *p* < 0.001) and final PE grade (r = 0.166, *p* = 0.001), but not with average grade in science subjects (r = −0.009, *p* = 0.851). Overall academic average was strongly correlated with science average (r = 0.809, *p* < 0.001) and moderately correlated with final PE grade (r = 0.474, *p* < 0.001). multivariate analysis of variance (MANOVA)revealed a significant but small overall difference in combined academic and PE performance across the four sleep-duration and physical-activity groups (Pillai’s Trace = 0.034, F(6, 870) = 2.522, *p* = 0.020, partial η^2^ = 0.017). Follow-up analyses showed no significant group differences in overall academic average (F(3, 435) = 1.460, *p* = 0.225, partial η^2^ = 0.010), whereas PE performance differed significantly across groups (F(3, 435) = 4.040, *p* = 0.007, partial η^2^ = 0.027). Conclusions: Sleep duration and physical activity showed weak but significant positive associations with overall academic average and PE performance among Moroccan adolescents, whereas neither was significantly associated with average grades in science subjects. Combined sleep-duration and physical-activity groups differed significantly in PE performance, but not in overall academic average, with small effect sizes. These findings suggest that sleep duration and physical activity are associated with adolescent performance, although the magnitude of these associations is modest and should not be interpreted as causal. Further longitudinal research using objective measures of sleep and physical activity is warranted to clarify these relationships.

## 1. Introduction

Sleep and physical activity are fundamental determinants of adolescent health, development, and educational success. During adolescence, a developmental period characterized by rapid biological, psychological, and social changes, lifestyle behaviors often diverge from recommended health guidelines, resulting in insufficient sleep and reduced engagement in physical activity. Such behavioral patterns may adversely affect cognitive functioning, emotional regulation, and physical performance, with potential consequences for school achievement and participation in Physical Education (PE) [1].

Sleep is widely recognized as a critical component of adolescent development. Adequate sleep supports neurocognitive processes such as attention, memory consolidation, executive functioning, and learning, all of which are essential for academic success and effective participation in school activities [1,2,3]. Conversely, insufficient sleep has been associated with impaired concentration, reduced motivation, slower recovery, and poorer academic outcomes. These effects highlight the potential relevance of sleep to both academic and physical performance during adolescence.

Physical activity represents another key lifestyle behavior associated with positive developmental outcomes. Regular participation in physical activity contributes to improved cardiovascular fitness, motor competence, psychological well-being, and self-regulation while also promoting cognitive functioning and academic achievement [4,5]. Furthermore, evidence suggests that physical activity and sleep are closely interconnected. Physical activity may improve sleep quality and duration through physiological and psychological mechanisms, whereas sufficient sleep may facilitate engagement in physical activity by enhancing energy levels, recovery, and motivation [6,7,8,9].

The present study is informed by a neurocognitive and behavioral framework that conceptualizes sleep and physical activity as interrelated determinants of adolescent performance. Neurocognitive models suggest that adequate sleep promotes cognitive efficiency, learning capacity, and emotional regulation, whereas behavioral health frameworks emphasize the role of physical activity in supporting physical fitness, motivation, and self-regulation. These mechanisms are particularly relevant in Physical Education, where successful performance depends not only on physical abilities but also on attention, motivation, decision-making, and behavioral engagement.

Previous research has consistently reported positive associations between sleep, physical activity, and various indicators of academic performance [4,5]. Studies examining adherence to integrated movement guidelines that combine adequate sleep and sufficient physical activity have also shown favorable effects on educational outcomes among adolescents [10,11]. Moreover, Ref. [12] demonstrated that the combined influence of sleep and physical activity contributes to academic achievement. However, most existing investigations have focused on general academic performance, cognitive outcomes, or health indicators rather than on Physical Education performance as a distinct educational domain.

This distinction is important because performance in Physical Education differs from general academic achievement. In the present study, physical activity refers to habitual engagement in bodily movement and exercise and is therefore distinct from Physical Education (PE) performance, which refers to students’ achievement in the school PE subject and is operationalized through their final PE grade. Accordingly, the terms “PE performance” and “final PE grade” refer to the same study outcome. Academic performance and academic achievement are used as broader concepts when referring to educational outcomes in the literature; in the present study, academic performance outside PE was specifically assessed using the average grade in science subjects, calculated from mathematics, physics–chemistry, and life and earth sciences. Unlike general academic indicators, PE performance may integrate physical fitness and motor competence with cognitive engagement, motivation, and active participation. Therefore, findings derived from traditional academic subjects may not be directly transferable to Physical Education settings. Despite growing interest in lifestyle behaviors and educational outcomes, the extent to which sleep duration and physical activity jointly contribute to PE performance remains insufficiently understood.

In addition, available evidence originates predominantly from Western populations. Research conducted in North African and Arab countries remains comparatively limited, despite indications that adolescents in these regions frequently experience insufficient sleep and low levels of physical activity [13]. Cultural, environmental, educational, and socioeconomic factors may influence lifestyle behaviors and their consequences, making it inappropriate to assume that findings from Western populations can be generalized to Moroccan adolescents. Consequently, context-specific investigations are needed to better understand how behavioral factors relate to educational outcomes in this population.

To address this gap, the present study examined the relationships between sleep duration, physical-activity level, and academic and Physical Education performance among Moroccan adolescents. Lifestyle behaviors were assessed using the Arab Teens Lifestyle Study (ATLS) questionnaire [14], a validated instrument widely used in Arab populations.

Based on the proposed theoretical framework and previous empirical findings, three hypotheses were formulated. First, adolescents reporting longer sleep duration were expected to demonstrate higher Physical Education performance scores. Second, higher levels of physical activity were expected to be associated with better Physical Education performance. Third, adolescents combining longer sleep duration with higher levels of physical activity were expected to exhibit the highest levels of Physical Education performance, reflecting the complementary contribution of these lifestyle behaviors to PE performance.

## 2. Results

### 2.1. Participants’ Characteristics

A total of 439 adolescents participated in the study. Girls represented 56.3% of the sample, whereas boys accounted for 43.7%. Most participants lived in urban areas (77.2%). Regarding educational level, the largest proportions were enrolled in the second (25.5%) and third (23.9%) years of middle school. Detailed sociodemographic characteristics of the participants are presented in Table 1.

### 2.2. Descriptive Characteristics of the Study Variables

Descriptive statistics for the study variables are presented in Table 2. The mean age of participants was 14.99 ± 2.06 years. Average sleep duration was 7.30 ± 1.95 h/night, whereas the mean physical-activity level was 1077.84 ± 701.01 MET-min/week. Boys reported higher levels of physical activity than girls, while girls showed slightly higher overall academic averages and average grades in science subjects.

### 2.3. Correlations Among Sleep Duration, Physical Activity, and Academic Variables

The distributions of the quantitative variables were assessed prior to correlation analysis using skewness and kurtosis. The skewness and kurtosis values were −1.174 and 0.315 for sleep duration, 1.973 and 6.315 for physical activity, −1.684 and 3.203 for PE grade, 0.036 and −1.122 for science average, and 0.079 and −0.882 for overall academic average, respectively. Most variables showed acceptable distributional characteristics, although elevated kurtosis values were observed for physical activity and PE grade. Pearson correlation coefficients were calculated to examine the associations between sleep duration, physical activity, and academic and Physical Education performance indicators. All correlation tests were two-tailed, and statistical significance was set at *p* < 0.05.

Pearson correlation coefficients among the study variables are presented in Table 3. Sleep duration was positively correlated with academic average (r = 0.147, *p* = 0.002) and Physical Education grade (r = 0.167, *p* = 0.001), whereas its correlations with science average (r = 0.080, *p* = 0.099) and physical activity (r = 0.010, *p* = 0.840) were not statistically significant. Physical activity was positively correlated with academic average (r = 0.169, *p* < 0.05) and Physical Education grade (r = 0.166, *p* = 0.001), but was not significantly correlated with science average (r = −0.009, *p* = 0.851). Academic average showed a strong positive correlation with science average (r = 0.809, *p* < 0.05) and a positive correlation with Physical Education grade (r = 0.474, *p* < 0.05). Science average was also positively correlated with Physical Education grade (r = 0.332, *p* < 0.05).

### 2.4. Descriptive Characteristics According to Sleep Duration and Physical-Activity Categories

Descriptive characteristics of academic and Physical Education performance across the four combined sleep-duration and physical-activity groups are presented in Table 4. The highest academic average was observed among participants sleeping >8 h/night and classified as active (14.35 ± 2.34), followed by active participants sleeping ≤8 h/night (14.22 ± 2.56). The lowest academic average was observed among participants sleeping >8 h/night and classified as inactive (13.48 ± 2.73). Similarly, the highest PE grade was observed in the >8 h and active group (17.72 ± 1.04), whereas the lowest PE grade was observed in the ≤8 h and inactive group (16.45 ± 2.61).

### 2.5. Differences in Academic and Physical Education Performance Across Sleep and Physical-Activity Groups

Prior to MANOVA, relevant assumptions were examined. The distributions of the dependent variables were assessed within each group using skewness and kurtosis. Overall academic average showed acceptable distributional characteristics across the four groups, whereas some departures from normality were observed for PE grade, particularly in the >8 h and inactive group. Multivariate outliers were assessed using Mahalanobis distance; the maximum observed value (9.277) was below the critical χ^2^ value of 13.82 for two dependent variables at *p* < 0.001, indicating no extreme multivariate outliers according to this criterion. Overall academic average and PE grade were moderately correlated (r = 0.474, *p* < 0.001), without evidence of excessive correlation between the dependent variables. Box’s M test was significant (*p* < 0.001), indicating heterogeneity of covariance matrices; therefore, Pillai’s Trace was retained for the multivariate interpretation. Levene’s test indicated homogeneity of variance for academic average (*p* = 0.250) but not for PE grade (*p* < 0.001). Accordingly, Games–Howell post hoc comparisons were used for PE performance.

The MANOVA revealed a statistically significant overall difference among the four combined sleep-duration and physical-activity groups in the combined academic and Physical Education performance outcomes (Pillai’s Trace = 0.034, F(6, 870) = 2.522, *p* = 0.020, partial η^2^ = 0.017). However, the magnitude of the multivariate effect was small (Table 5).

Follow-up univariate analyses were conducted to examine whether academic and PE performance differed across the four combined sleep-duration and physical-activity groups (Table 6). No statistically significant difference was observed for overall academic average (F(3, 435) = 1.460, *p* = 0.225, partial η^2^ = 0.010). In contrast, PE performance differed significantly across the four groups (F(3, 435) = 4.040, *p* = 0.007, partial η^2^ = 0.027), although the effect size was small. Levene’s test indicated that the assumption of homogeneity of variances was met for overall academic average (F(3, 435) = 1.375, *p* = 0.250) but not for PE performance (F(3, 435) = 11.411, *p* < 0.001).

### 2.6. Post Hoc Comparisons

Because the homogeneity-of-variance assumption was violated for PE performance, Games–Howell post hoc comparisons were conducted to identify differences between the four combined sleep-duration and physical-activity groups. PE grades were significantly higher in the >8 h and active group than in the ≤8 h and inactive group (mean difference = 1.275, 95% CI [0.591, 1.960], *p* < 0.001). In addition, among participants sleeping ≤8 h/night, the active group had significantly higher PE grades than the inactive group (mean difference = 0.736, 95% CI [0.030, 1.441], *p* = 0.037). No other pairwise differences in PE performance were statistically significant (*p* > 0.05) (Table 7).

## 3. Discussion

This study aimed to examine the relationships between sleep duration, physical activity, and academic and physical performance among Moroccan adolescents. Overall, the findings indicate that sleep duration and physical activity were positively associated with overall academic average and Physical Education performance, although the observed correlations were weak. Neither sleep duration nor physical activity was significantly associated with the average grade in science subjects. Furthermore, the combined sleep-duration and physical-activity groups differed significantly in PE performance, but not in overall academic average. These findings support the view that adolescent performance is influenced by multiple interacting behavioral, cognitive, and environmental factors rather than by a single determinant [15].

Moreover, beyond behavioral factors, previous studies have demonstrated that psychological variables, such as motivation and mental skills, play a crucial role in performance in Physical Education, further reinforcing the multifactorial and multidimensional nature of adolescent performance [16].

The correlation analysis revealed that significant associations were primarily observed between academic variables, particularly between academic average and science average, as well as between academic average and Physical Education grades. These findings suggest a degree of consistency across different indicators of school achievement. Physical activity was weakly but significantly associated with overall academic average (r = 0.169, *p* < 0.001) and Physical Education grade (r = 0.166, *p* = 0.001), whereas no significant association was observed with science average (r = −0.009, *p* = 0.851). Although several studies have reported positive relationships between physical activity and academic achievement, the evidence remains heterogeneous, and contextual, cultural, and methodological differences may contribute to inconsistent findings [17,18,19].

Sleep duration showed small but significant correlations with overall academic average (r = 0.147, *p* = 0.002) and Physical Education grade (r = 0.167, *p* = 0.001), whereas its association with science average was not statistically significant (r = 0.080, *p* = 0.099). These findings are consistent with previous research indicating that sleep duration is positively associated with academic outcomes, although the strength of this relationship is generally modest [2,20]. Adequate sleep may support attention, memory consolidation, learning processes, and emotional regulation, which are important components of academic functioning. However, the relatively small magnitude of the observed significant correlations (r = 0.147–0.167) suggests limited practical significance and indicates that sleep duration represents only one of several factors associated with educational outcomes. Accordingly, these associations should be interpreted as population-level statistical tendencies rather than deterministic relationships at the individual level. This distinction between population-level associations and individual outcomes has also been emphasized in longitudinal research in other behavioral contexts, where statistically significant associations may coexist with substantial inter-individual variability [21]. In the present study, considerable inter-individual variability may similarly exist, and sleep duration alone cannot be used to predict an individual adolescent’s academic or PE performance. Academic performance is a multifactorial outcome that may reflect the combined influence of sleep characteristics, individual sleep need, physical activity, mental health, family and socioeconomic conditions, learning habits, screen-use patterns, and other individual and environmental factors.

The multivariate analysis revealed a statistically significant overall difference among the four combined sleep-duration and physical-activity groups in academic and PE performance (Pillai’s Trace = 0.034, F(6, 870) = 2.522, *p* = 0.020, partial η^2^ = 0.017), although the magnitude of the multivariate effect was small. Follow-up analyses showed no significant differences in overall academic average across the four groups (F(3, 435) = 1.460, *p* = 0.225, partial η^2^ = 0.010). In contrast, PE performance differed significantly across groups (F(3, 435) = 4.040, *p* = 0.007, partial η^2^ = 0.027), although this effect was also small. Games–Howell post hoc comparisons indicated that PE grades were significantly higher in the >8 h and active group than in the ≤8 h and inactive group (mean difference = 1.275, *p* < 0.001). Among adolescents sleeping ≤8 h/night, active participants also had significantly higher PE grades than inactive participants (mean difference = 0.736, *p* = 0.037). No other pairwise differences in PE performance were statistically significant. However, this finding should be interpreted cautiously. Categorizing continuous behavioral variables may reduce statistical sensitivity and obscure gradual associations that could be detected using continuous measures. The small effect sizes observed in both the multivariate and univariate analyses indicate that the combined sleep-duration and physical-activity grouping explained only a limited proportion of variability in performance outcomes. Therefore, the observed group differences in PE performance should not be interpreted as evidence of a deterministic or causal effect of sleep duration or physical activity on performance. Previous research examining the combined effects of sleep and physical activity has also highlighted the difficulty of detecting clear group-based differences, particularly when behavioral patterns overlap [12].

Sex differences observed in this study, with boys reporting higher levels of physical activity and girls showing slightly higher Physical Education grades, may be explained by differences in access to sports opportunities, participation in organized physical activities, and gender-related social norms. Previous studies have consistently reported lower levels of physical activity among adolescent girls, often linked to sociocultural and environmental constraints [22]. Nevertheless, the present study was not designed to examine whether sex moderates the relationships between sleep duration, physical activity, and performance outcomes. Future research should therefore investigate potential sex-specific associations using more advanced analytical approaches.

It is important to emphasize that the present study focused exclusively on sleep duration and did not assess other dimensions of sleep health, such as sleep quality, sleep efficiency, bedtime and wake time, sleep regularity, social jetlag, weekend catch-up sleep, daytime sleepiness, or sleep disorders, which may also influence cognitive functioning and academic performance [23]. Consequently, interpretations should be limited to sleep duration rather than sleep health as a whole.

Furthermore, absolute sleep duration may not fully reflect sleep adequacy in adolescents, as individual sleep need can vary between individuals [24]. Therefore, the same reported sleep duration may not necessarily represent the same degree of sleep sufficiency for all adolescents. The present study did not assess individual sleep need or optimal sleep duration. Experimental research has also attempted to estimate sleep need in adolescents and has shown that optimal sleep duration may depend on the functional outcome considered [25]. Future studies should therefore consider individual variability in sleep need alongside conventional age-based sleep-duration recommendations.

Another important consideration concerns the use of Physical Education grades as a proxy for physical performance. Although commonly used in school-based research, these grades may reflect not only physical competence but also behavioral factors such as effort, participation, attendance, and teacher evaluation criteria. Despite this limitation, Physical Education grades remain a practical and ecologically valid indicator of student achievement within the school environment. This limitation should nevertheless be considered when interpreting the associations involving Physical Education performance.

Several limitations should be acknowledged. First, the cross-sectional design precludes causal inferences regarding the relationships between sleep duration, physical activity, and academic performance. Second, sleep duration was assessed using a single self-reported item, which does not capture the multidimensional nature of sleep and may be affected by recall bias. Third, physical activity was also measured using self-reported data rather than objective measures such as accelerometry, which may introduce reporting inaccuracies. Fourth, important confounding variables, such as socioeconomic status, family environment, dietary habits, screen time, mental health, and pubertal stage, were not controlled for, although these factors may influence both lifestyle behaviors and educational outcomes. Finally, the use of convenience sampling and the predominance of female and urban participants may limit the generalizability of the findings. These limitations are consistent with those reported in similar epidemiological studies [5].

Despite these limitations, the findings have practical implications. Adequate sleep duration and physical activity may represent components of a broader pattern of lifestyle factors associated with adolescent well-being and educational and PE outcomes. However, given the weak correlations and small effect sizes observed, sleep duration and physical activity should be considered as components of a broader set of determinants rather than primary drivers of adolescent performance. Similar patterns have been observed in Moroccan adolescents, highlighting the role of lifestyle behaviors within a wider framework of factors influencing educational achievement [26].

## 4. Materials and Methods

### 4.1. Study Design

This study employed an analytical cross-sectional design, conducted in March 2024 in the city of Oued Zem, located in the Beni Mellal–Khenifra region of Morocco. A cross-sectional approach was selected to investigate the relationships between lifestyle behaviors and educational outcomes among adolescents at a specific point in time.

### 4.2. Participants

A total of 439 adolescents (boys and girls), aged 14–20 years, enrolled in five public middle and high schools participated in the study. Participants were recruited using a convenience sampling strategy based on school accessibility and logistical feasibility. A total of 540 questionnaires were distributed across the five participating schools, of which 480 were returned, corresponding to a response rate of 88.9%. After screening the questionnaires for completeness and consistency, 439 were retained for the final analysis.

Eligibility criteria included regular attendance in Physical Education (PE) classes and voluntary participation in the study. Students reporting chronic health conditions that could affect physical activity participation or those with frequent absences from PE classes were excluded.

### 4.3. Procedures

Data collection was carried out in March 2024 in collaboration with several public secondary schools. Prior to data collection, school administrators, Physical Education teachers, students, and parents were informed about the objectives and procedures of the study.

Questionnaires were administered collectively during regular school hours in classroom settings under teacher supervision to ensure standardized conditions. Participation was voluntary and anonymous. Written informed consent was obtained from both the students and their parents or legal guardians before participation.

To minimize potential sources of bias, data collection was conducted outside examination periods. The study was conducted in accordance with the ethical principles outlined in the Declaration of Helsinki [27].

### 4.4. Instruments

Data were collected using a structured self-administered questionnaire based on the Arab Teens Lifestyle Study (ATLS) protocol. The ATLS questionnaire has demonstrated good reliability (intraclass correlation coefficient [ICC] = 0.85) and acceptable validity when compared with objective measures of physical activity among adolescents [28].

#### 4.4.1. Physical Activity

Physical activity was assessed through self-reported frequency, duration, and intensity of weekly activities. Reported activities were converted into metabolic equivalent task values (METs) using the Compendium of Physical Activities [29] and the Youth Compendium of Physical Activities [30].

Total physical activity was calculated as MET-minutes per week using the following formula:

MET-min/week = MET × duration (minutes) × frequency (days/week)

Physical activity was initially quantified as a continuous variable expressed in MET-minutes per week. For descriptive and interpretative purposes, participants were subsequently categorized as active (≥1680 MET-min/week) or inactive (<1680 MET-min/week), corresponding approximately to the international recommendation of at least 60 min of moderate-to-vigorous physical activity per day for adolescents [31].

Although this categorization facilitates comparison with public health recommendations, it may reduce statistical sensitivity by simplifying the variability of physical activity behaviors.

#### 4.4.2. Sleep Duration

Sleep duration was assessed using an item derived from the Arab Teens Lifestyle Study (ATLS) questionnaire. Participants were asked to report their average daily sleep duration, including both nighttime sleep and daytime naps during a typical week [28].

Self-reported sleep measures are commonly used in large-scale epidemiological studies because of their practicality and feasibility in school-based settings. However, such measures may be affected by recall bias and do not capture other dimensions of sleep, such as sleep quality, sleep efficiency, or sleep timing.

Prior to analysis, questionnaire responses were screened for completeness and consistency. Participants with missing sleep-duration data were excluded from the final analyses.

#### 4.4.3. Academic and Physical Education Performance Measures

Academic performance data were obtained from official school records. Three indicators were retained: the overall academic average, the average grade in science subjects (mathematics, physics–chemistry, and life and earth sciences), and the final grade in Physical Education.

In the Moroccan public education system, Physical Education grades are assigned according to a national curriculum and assessment framework established by the Ministry of National Education. These grades reflect several dimensions of student achievement, including motor performance, participation, effort, engagement, and behavioral involvement during PE classes.

Although PE grades do not represent a pure measure of physical competence, they provide an ecologically valid indicator of achievement within the educational setting and have been used in previous educational research. Nevertheless, variability in grading practices across teachers and schools cannot be completely excluded and should be considered when interpreting the findings.

### 4.5. Statistical Analysis

Statistical analyses were performed using IBM SPSS Statistics software version 22.0.0.0. Descriptive statistics, including means, standard deviations, frequencies, and percentages, were calculated to summarize participants’ characteristics and study variables. The distributions of quantitative variables were examined using skewness and kurtosis. Pearson correlation coefficients were calculated to examine the associations between sleep duration, physical activity, overall academic average, average grade in science subjects, and Physical Education (PE) grade. All correlation tests were two-tailed.

Participants were recoded into four groups according to combined sleep-duration (>8 h/night vs. ≤8 h/night) and physical-activity (active vs. inactive) categories. A multivariate analysis of variance (MANOVA) was conducted to examine differences in overall academic average and PE grade across these four groups. Prior to MANOVA, relevant assumptions were examined. Distributional characteristics of the dependent variables were assessed within each group using skewness and kurtosis, and potential multivariate outliers were evaluated using Mahalanobis distance. The relationship between the dependent variables was examined using Pearson correlation. Homogeneity of covariance matrices was assessed using Box’s M test, and homogeneity of variances was evaluated using Levene’s test. Because Box’s M test indicated heterogeneity of covariance matrices, Pillai’s Trace was retained for interpretation of the multivariate effect. Follow-up univariate analyses were conducted for each dependent variable, and effect sizes were reported using partial eta squared (partial η^2^). Because the homogeneity-of-variance assumption was violated for PE grade, Games–Howell post hoc comparisons were used to examine pairwise group differences in PE performance. Statistical significance was set at *p* < 0.05.

### 4.6. Ethical Considerations

The study was conducted in accordance with the ethical principles of the Declaration of Helsinki [27].

Authorization to conduct the study was obtained from the Provincial Directorate of the Ministry of National Education and from the administrations of the participating schools.

Because this study involved an anonymous, non-interventional, cross-sectional survey conducted in an educational setting and did not involve clinical procedures, biological sampling, or the collection of sensitive personal data, formal approval from an institutional ethics committee was not required under the administrative procedures governing school-based research within the local educational system.

Participation was voluntary. Written informed consent was obtained from both students and their parents or legal guardians prior to participation. All collected data were anonymized and treated confidentially throughout the study.

## 5. Conclusions

This study examined the relationships between sleep duration, physical activity, and academic and Physical Education performance among Moroccan adolescents. The findings revealed weak but significant positive associations of both sleep duration and physical activity with overall academic average and Physical Education performance, whereas neither behavior was significantly associated with the average grade in science subjects. In addition, the combined sleep-duration and physical-activity groups differed significantly in Physical Education performance, but not in overall academic average, with small effect sizes.

Overall, the findings provide only partial support for the study hypotheses and suggest that sleep duration and physical activity are associated with adolescent performance, although the magnitude of these relationships is modest and varies according to the performance indicator considered. However, the observed associations were generally small, indicating that sleep duration and physical activity represent only part of a broader set of factors influencing educational outcomes.

These findings should be interpreted with caution given the cross-sectional design, the use of self-reported measures, and the absence of several potentially relevant confounding variables.

Future research should investigate whether sex moderates the relationships between sleep duration, physical activity, and educational outcomes. Longitudinal studies incorporating objective assessments of sleep (e.g., actigraphy) and physical activity (e.g., accelerometry), together with multidimensional measures of sleep health, may provide a more comprehensive understanding of these associations. Future research should also consider individual variability in sleep need and explore approaches for estimating individual optimal sleep duration rather than relying exclusively on absolute sleep duration.

## Figures and Tables

**Table 1:** Sociodemographic characteristics of participants (*n* = 439).

Variable	Frequency	Percentage %
Gender		
Male	192	43.70
Female	247	56.30
Age		
14 years old	222	50.56
15 to 17 years old	157	35.77
18 to 20 years old	60	13.67
Area of residence		
Urban	339	77.20
Rural	100	22.80
Education level		
Year 2 of middle school	198	45.10
Year 3 of middle school	105	23.90
10th grade	24	5.50
1st year of high school A	26	5.90
1st year of high school B	36	8.20
2nd year of high school A	24	5.50
2nd year of high school B	26	5.90

Values are presented as frequency (*n*) and percentage (%).

**Table 2:** Descriptive characteristics of the study variables according to sex.

Variable	Total (Mean ± SD)	Boys (Mean ± SD)	Girls (Mean ± SD)
Age (years)	14.99 ± 2.06	15.05 ± 2.04	14.95 ± 2.08
Sleep duration (h/night)	7.30 ± 1.95	7.26 ± 1.99	7.33 ± 1.93
PA (MET-min/week)	1077.84 ± 701.01	1315.54 ± 877.98	893.08 ± 445.06
Sci. avg (/20)	13.31 ± 3.37	12.85 ± 3.34	13.68 ± 3.36
PE (/20)	16.75 ± 2.35	16.69 ± 2.27	16.80 ± 2.41
Acad. avg (/20)	13.63 ± 2.76	13.16 ± 2.77	14.01 ± 2.70

Values are presented as mean ± standard deviation (SD). Legend: SD—standard deviation; MET-min/week—metabolic equivalent minutes per week; PE—Physical Education; Acad. avg—overall academic average; Sci. average—average grade in science subjects.

**Table 3:** Pearson correlation coefficients among study variables.

Variables	Sleep Duration (h/night)	Acad. Avg (/20)	PA (MET-min/week)	Sci. Avg (/20)	PE (/20)
Sleep duration (h/night)	1	0.147	0.010	0.080	0.167
Acad. avg (/20)	0.147	1	0.169	0.809	0.474
PA (MET-min/week)	0.010	0.169	1	−0.009	0.166
Sci. avg (/20)	0.080	0.809	−0.009	1	0.332
PE (/20)	0.167	0.474	0.166	0.332	1

Legend: PA-physical activity; PE-Physical Education; acad. avg-overall academic average; sci.avg-average grade in science subjects. *p* < 0.05.

**Table 4:** Academic and Physical Education performance according to combined sleep-duration and physical-activity categories.

Group	*n*	Acad. Avg (/20)	PE (/20)
>8 h & Active	27	14.35 ± 2.34	17.72 ± 1.04
>8 h & Inactive	120	13.48 ± 2.73	17.03 ± 2.13
≤8 h & Active	41	14.22 ± 2.56	17.18 ± 1.36
≤8 h & Inactive	251	13.54 ± 2.84	16.45 ± 2.61

Legend: PE—Physical Education; acad.Avg-overall academic average. Values are presented as mean ± SD.

**Table 5:** Multivariate analysis of academic and Physical Education performance across combined sleep-duration and physical-activity groups.

Effect	Multivariate Test	Value	F	df	Error df	*p*-Value	Partial η^2^
Combined sleep–PA group	Pillai’s Trace	0.034	2.522	6	870	0.020	0.017

Note: PA, physical activity; PE, Physical Education. The four groups were defined according to combined sleep-duration and physical-activity categories. Box’s M test was significant (M = 47.478, F = 5.181, *p* < 0.001), indicating heterogeneity of the covariance matrices; therefore, Pillai’s Trace was used for interpretation.

**Table 6:** Univariate effects of combined sleep-duration and physical-activity groups on academic and Physical Education performance.

Outcome	F	df	*p*-Value	Partial η^2^
Overall academic average	1.460	3, 435	0.225	0.010
PE grade	4.040	3, 435	0.007	0.027

Note: PE, Physical Education. Results represent follow-up univariate tests for the four combined sleep-duration and physical-activity groups. Statistical significance was set at *p* < 0.05.

**Table 7:** Games–Howell post hoc comparisons of Physical Education performance across combined sleep-duration and physical-activity groups.

Group Comparison	Mean Difference	SE	95% CI	*p*-Value
>8 h & Active vs. >8 h & Inactive	0.692	0.280	−0.042 to 1.426	0.072
>8 h & Active vs. ≤8 h & Active	0.540	0.293	−0.233 to 1.312	0.263
>8 h & Active vs. ≤8 h & Inactive	1.275	0.260	0.591 to 1.960	<0.001
>8 h & Inactive vs. ≤8 h & Active	−0.152	0.289	−0.906 to 0.602	0.953
>8 h & Inactive vs. ≤8 h & Inactive	0.584	0.256	−0.077 to 1.244	0.104
≤8 h & Active vs. ≤8 h & Inactive	0.736	0.270	0.030 to 1.441	0.037

Note: SE, standard error; CI, confidence interval. Mean differences are expressed in PE grade points (/20). Games–Howell post hoc comparisons were used because the homogeneity-of-variance assumption was violated for PE performance (Levene’s test, *p* < 0.001). Statistically significant comparisons are shown in bold (*p* < 0.05).

## Data Availability

The data presented in this study are available from the corresponding author upon reasonable request.

## Abbreviations

ATLS	Arab Teens Lifestyle Study
CI	Confidence Interval
ICC	Intraclass Correlation Coefficient
MET	Metabolic Equivalent of Task
MET-min/week	Metabolic Equivalent Minutes per Week
MS	Mean Square
OECD	Organisation for Economic Co-operation and Development
PA	Physical Activity
PE	Physical Education
WHO	World Health Organization
